# *CDKN2AIPNL*: a potential pan-cancer biomarker

**DOI:** 10.3389/fgene.2025.1588292

**Published:** 2026-01-21

**Authors:** Yulin Yuan, Sheng-Xiao Ma, Heshi Liu, Yang Gong, Xuan Sun, Quan Wang, Weifu Zhang

**Affiliations:** 1 Maoming Hospital of Traditional Chinese Medicine, Maoming, Guangdong, China; 2 Department of Gastrocolorectal Surgery, The First Hospital of Jilin University, Changchun, China

**Keywords:** *CDKN2AIPNL*, metabolic reprogramming, pan-cancer, prognostic biomarker, tumor microenvironment

## Abstract

Cancer progression involves dynamic crosstalk between tumor-intrinsic pathways and microenvironmental remodeling, and identifying pan-cancer biomarkers is critical for precision oncology. *CDKN2AIPNL* exhibits a paradoxical role in cancer, acting as a tumor suppressor in myeloid malignancies but promoting solid tumor progression, yet its systematic pan-cancer characteristics remain unelucidated. This study aimed to comprehensively analyze *CDKN2AIPNL’s* expression patterns, prognostic value, genetic alterations, and molecular mechanisms across multiple tumor types using public datasets including TCGA, GTEx, HPA, and tools such as GEPIA2, cBioPortal, TIMER2, STRING, and BioGRID. We performed expression difference analysis, survival analysis (overall survival, disease-free survival, progression-free survival), genetic alteration analysis, cancer-associated fibroblast (CAF) infiltration analysis, and gene/protein interaction enrichment analysis. Results showed that CDKN2AIPNL was significantly upregulated in multiple tumors (e.g., LIHC, UVM, BRCA, LUAD) and downregulated in others (e.g., KICH, KIRP, THCA), with high tumor specificity. Elevated CDKN2AIPNL expression correlated with poor overall survival in LIHC (HR = 1.7, p = 0.0026), UVM (HR = 26, p = 2.2e-6), BRCA (HR = 26, p = 2.2e-6), LUAD (HR = 1.36, p = 0.049), PCPG (HR = 1.71, p = 0.0012), and TGCT (HR = 0.37, p=0.023), and was associated with advanced tumor stages in metabolically active cancers. Genetic alterations (amplifications and mutations) were frequent in KIRC (>5%) and ACC (>4%), with all mutations localized to the XTBD region, and amplification predicted poor prognosis in PRAD (p = 0.008) while mutations conferred favorable outcomes in BLCA. CDKN2AIPNL expression positively correlated with CAF infiltration in ESCA, KICH, UVM, and other tumors, and interacted with MYC, XRN2, and CHAMP1 to regulate metabolic reprogramming, cell cycle, and immune suppression. Our findings systematically reveal CDKN2AIPNL’s dual role in tumorigenesis and validate it as a potential pan-cancer prognostic biomarker, providing novel insights for cancer diagnosis and targeted therapy.

## Introduction

According to data from the American Cancer Society, an estimated 611,720 people in the United States will die from cancer in 2024 (Siegel et al., 2024). Considering the population aging, this number is expected to increase further. Although targeted therapies and immunotherapies have demonstrated significant efficacy in treating various cancers, clinical outcomes exhibit substantial variability across different cancer types (Waldman et al., 2020; Xiao et al., 2023; Sonkin et al., 2024). Therefore, to broaden potential treatment options for malignant tumors, it is imperative to enhance our understanding of oncogenesis and tumor progression through the identification of oncogenes.

*CDKN2A* interacting protein N-terminal like (*CDKN2AIPNL*) is a homolog of the N-terminal domain of a protein that interacts with the *CDKN2A* gene. The *CDKN2AIPNL* gene is situated at position 5q31.1 on human chromosome 5, comprises three exons, and encodes a protein that is active in the nucleolus and nucleoplasm (details at https://www.ncbi.nlm.nih.gov/gene/91368#gene-expression). *CDKN2AIPNL* is expressed in a range of normal tissues, with particularly high expression levels in the thyroid and testis, and it is frequently classified as a tumor suppressor gene (Razvi et al., 2018). *CDKN2AIPNL* exhibits a paradoxical role in cancer progression: while acting as a tumor suppressor in myeloid malignancies by maintaining genomic stability (Visconte et al., 2017), it promotes tumor progression in solid cancers via metabolic reprogramming and immune evasion (Liu et al., 2019; Azimi et al., 2023). This functional duality underscores the need for systematic pan-cancer analyses.

*CDKN2AIPNL* is associated with the activity and stability of the 5′–3′ exoribonuclease *XRN2* (Alexandrova et al., 2020). Research has demonstrated that *CDKN2AIPNL* is involved in regulating nucleic acid metabolism in colon cancer (Chodary Khameneh et al., 2022). Nonetheless, because *CDKN2AIPNL* remains a relatively understudied gene, its specific functions and mechanisms of action are not yet fully elucidated.

In this study, we systematically analyzed the expression status, prognostic value, genetic alterations, and molecular functions of *CDKN2AIPNL*, as well as its correlation with cancer-associated fibroblast infiltration across various tumor types.

## Materials and methods

### Gene expression analysis

To construct an mRNA expression map for *CDKN2AIPNL*, we utilized the Human Protein Atlas (HPA) database (https://www.proteinatlas.org/). We employed the “Gene DE” module of the Tumor Immune Estimation Resource (TIMER2) (http://timer.cistrome.org/) to evaluate the expression differences of *CDKN2AIPNL* between tumor and non-tumor tissues across various cancer types. In this module, we also examined *CDKN2AIPNL* expression across different molecular subgroups of breast cancer, as well as between HPV-positive and HPV-negative head and neck squamous cell carcinoma (HNSC) and between primary and metastatic skin cutaneous melanoma (SKCM). Additionally, we retrieved expression data for *CDKN2AIPNL* from the GEPIA2 database (http://gepia2.cancer-pku.cn/) to provide comparative validation of its expression levels. (Statistical significance was defined as *p* < 0.05).

### Survival analysis

Overall survival (OS) and disease-free survival (DFS) Kaplan-Meier (KM) plots, along with survival significance plots for *CDKN2AIPNL* across all TCGA tumor types, were generated using the “Survival Analysis” module of GEPIA2 (http://gepia2.cancer-pku.cn/). Furthermore, the UCSC Xena browser (https://xenabrowser.net/), with high/low expression groups defined by median cutoffs was used to perform progression-free survival (PFS) analysis of *CDKN2AIPNL* using the TCGA Pan-Cancer dataset. The Kaplan-Meier plotter database was used to augment the prognostic analysis results. The expression threshold for high and low *CDKN2AIPNL* expression was set at 50%. For survival analysis, p-values were adjusted using the Benjamini–Hochberg method to control the false discovery rate (FDR) across multiple comparisons.

### Genetic alteration analysis

Genetic alterations of *CDKN2AIPNL* were analyzed using cBioPortal (https://www.cbioportal.org/). Utilizing the TCGA Pan-Cancer Atlas study dataset, we calculated the frequency of *CDKN2AIPNL* gene mutations and copy number alterations via the “Cancer Type Summary” module. Additionally, the “Mutations” module was employed to create a mutation map for *CDKN2AIPNL*.

To assess the correlation between *CDKN2AIPNL* amplification status and prognosis in Prostate Adenocarcinoma (PRAD), molecular profiles were selected based on copy number alterations, and cases were divided into altered and unaltered groups to generate survival plots.

Similarly, for bladder urothelial carcinoma (BLCA), we evaluated the correlation between *CDKN2AIPNL* mutation status and prognosis by selecting molecular profiles based on mutations and categorizing cases into altered and unaltered groups to generate survival plots.

### Immune cell infiltration analysis

The “Immune” module of TIMER2 (http://timer.cistrome.org/) was employed to investigate the correlation between *CDKN2AIPNL* expression and cancer-associated fibroblast infiltration using the Extended Multi-Dimensional Immune Profiling (EPIC) and Tumor Immune Dysfunction and Exclusion (TIDE) algorithms.

### *CDKN2AIPNL*-related gene enrichment analysis

The STRING tool (https://string-db.org/) was utilized to construct a co-expression network for *CDKN2AIPNL* in *Homo sapiens*, employing the following parameters: (1) Active interaction sources: co-expression; (2) Meaning of network edges: evidence; (3) Maximum number of interactors: 50; and (4) Minimum required interaction score: low confidence (0.150).

The “Similar Gene Detection” module of GEPIA2 was used to extract 100 genes related to *CDKN2AIPNL* from the TCGA dataset, which have the most similar expression patterns with *CDKN2AIPNL*. The gene symbols of these 100 related genes were subsequently input into the ‘clusterProfiler’ R package for gene ontology (GO) pathway enrichment analysis, allowing for the identification of significantly enriched biological processes and pathways. Additionally, the ‘Correlation Analysis’ module of GEPIA2 facilitated pairwise gene correlation analysis, providing insights into the co-expression patterns of *CDKN2AIPNL* and its related genes.

### *CDKN2AIPNL*-protein interaction analysis

The “Network” module of BioGRID (https://thebiogrid.org/) was used to create a *CDKN2AIPNL*-protein interaction network, with the layout set to “Concentric circles.”

### Conservation status analysis of *CDKN2AIPNL*

The UCSC Genome Browser (http://www.genome.ucsc.edu/cgi-bin/hgTracks) was utilized to visualize the gene conservation of *CDKN2AIPNL* among vertebrates.

## Results

### Gene expression analysis of *CDKN2AIPNL*

**FIGURE 1:**
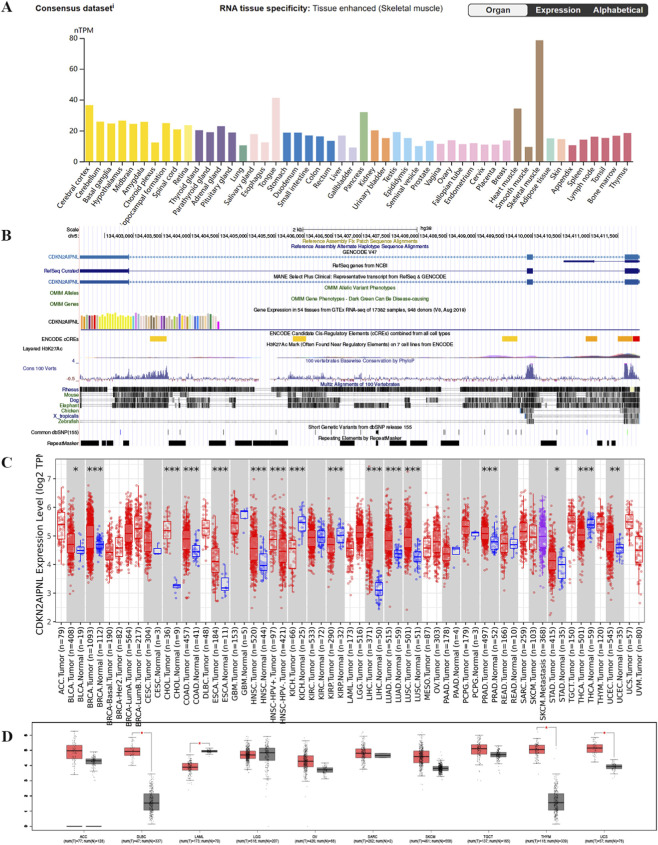
Expression status of *CDKN2AIPNL* in different tumors and normal tissues and gene conservation of *CDKN2AIPNL* in vertebrates. **(A)** Tissue expression consensus *CDKN2AIPNL* based on HPA, GTEx, and FANTOM5 datasets. **(B)** Gene conservation analysis of *CDKN2AIPNL* among vertebrates visualized using UCSC genome browser. **(C)** Expression status of *CDKN2AIPNL* in different tumor types visualized through TIMER2. *p < 0.05; **p < 0.01; p < 0.001. **(D)** Expression of *CDKN2AIPNL* in several cancers and paired normal tissues in the GEPIA database.

Comprehensive analysis of datasets from the Human Protein Atlas (HPA), Genotype-Tissue Expression (GTEx), and FANTOM5 (Functional Annotation of Mammalian Genomes) demonstrated that *CDKN2AIPNL* is highly expressed in metabolically active tissues, including skeletal muscle, cardiac muscle, pancreas, and brain tissue, with a particularly pronounced expression in skeletal muscle (Figure 1A; Supplementary Figure S1). Additionally, single-cell RNA sequencing data further corroborated these findings, revealing significant expression of *CDKN2AIPNL* in cardiomyocytes, skeletal muscle cells, and smooth muscle cells (Figure 1A; Supplementary Figure S1). These observations suggest that *CDKN2AIPNL* exhibits low tissue specificity, indicating a broad functional role across various tissues. Furthermore, our analysis revealed that *CDKN2AIPNL* is relatively conserved among vertebrate species (Figure 1B), which underscores its potential functional significance across evolutionary contexts.

We then investigated the expression pattern of *CDKN2AIPNL* in tumor tissues and found that the mRNA expression of *CDKN2AIPNL* exhibited high tumor specificity. The high tumor specificity of *CDKN2AIPNL* is not only beneficial for early screening of neoplastic diseases, improving the accuracy of early diagnosis and enabling earlier treatment for patients, but also makes *CDKN2AIPNL* a potential target for drug development. Notably, compared with the corresponding normal tissues, *CDKN2AIPNL* mRNA levels were significantly elevated in several tumor types (Figure 1C). Specifically, tumor tissues from Breast invasive carcinoma (BRCA)(T:N = 1093:112), Cholangiocarcinoma (CHOL)(T:N = 36:9), Colon adenocarcinoma (COAD)(T:N = 457:41), Esophageal carcinoma (ESCA)(T:N = 184:11), Head and Neck squamous cell carcinoma (HNSC)(T:N = 520:44), Liver hepatocellular carcinoma (LIHC)(T:N = 371:50), Lung adenocarcinoma (LUAD)(T:N = 515:59), Lung squamous cell carcinoma (LUSC)(T:N = 501:51), PRAD (T:N = 497:52), Uterine Corpus Endometrial Carcinoma (UCEC)(T:N = 545:35), and BLCA (T:N = 408:19) exhibited significantly higher *CDKN2AIPNL* expression compared to their respective normal tissues (Figure 1C). Furthermore, HPV-positive HNSC(T:N = 520:44) tissues showed markedly elevated *CDKN2AIPNL* expression compared to HPV-negative tissues (Figure 1C). Conversely, significantly reduced *CDKN2AIPNL* expression was noted in Kidney Chromophobe (KICH)(T:N = 66:25), Kidney Renal Papillary Cell Carcinoma (KIRP)(T:N = 290:32), and Thyroid Carcinoma (THCA)(T:N = 501:59) tumor tissues (Figure 1C).

To further elucidate the expression differences of *CDKN2AIPNL* in cancers lacking paired normal tissues, we utilized the GEPIA database, as indicated by the TIMER database. Figure 1D shows elevated *CDKN2AIPNL* expression in Lymphoid Neoplasm Diffuse Large B-cell Lymphoma (DLBC)(T:N = 47:337) (*p* = 0.003), Uterine Carcinosarcoma (UCS)(T:N = 57:78) (*p* = 0.004), reduced expression in Acute Myeloid Leukemia (LAML)(T:N = 173:70) (*p* = 0.01). Advanced tumor stages in LIHC and PAAD showed significantly elevated *CDKN2AIPNL* expression (*p* < 0.05, Figure 4), suggesting its role in malignant progression.

In summary, these findings suggest that *CDKN2AIPNL* may promote oncogenesis across various tumor types, highlighting the need for further investigation into its clinical significance.

### Correlation between *CDKN2AIPNL* expression and cancer patient prognosis

**FIGURE 2:**
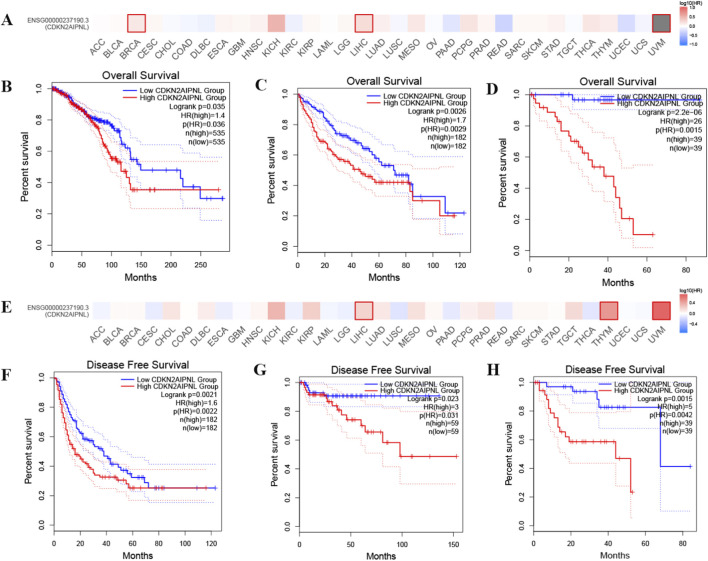
Correlation between *CDKN2AIPNL* expression and disease-free survival and overall survival in patients with different TCGA tumor types in the GEPIA2 database. GEPIA2 was used to construct survival plots **(A,E)** and perform OS **(B–D)** and DFS **(F–H)** analysis. Survival plots and Kaplan-Meier plots with significant results are displayed. The 95% confidence intervals for disease-free survival and overall survival are represented by red and blue dashed lines for the high *CDKN2AIPNL* and low groups, respectively.

To explore the potential prognostic value of *CDKN2AIPNL* based on the TCGA dataset, we employed the GEPIA2 and Kaplan-Meier plotter database to analyze the correlation between *CDKN2AIPNL* expression and prognosis in patients with different tumors (Figures 2A,E). In the GEPIA2, we found that lower *CDKN2AIPNL* expression was significantly associated with longer OS and DFS in LIHC (n = 364, OS: *HR =* 1.7, *P =* 0.0026; n = 364, DFS: *HR =* 1.6, *P =* 0.0021, Figures 2C,F) and UVM (n = 78, OS: *HR =* 26, *P =* 2.2e-6; n = 78, DFS: *HR =* 5, *P =* 0.0015, Figures 2D,H). In BRCA, lower *CDKN2AIPNL* expression was correlated with improved OS (n = 78, *HR =* 26, *P =* 2.2e-6, Figure 2B). Similarly, in THYM, lower *CDKN2AIPNL* expression was associated with better DFS (n = 118, *HR =* 3, *P =* 0.023, Figure 2G).

**FIGURE 3:**
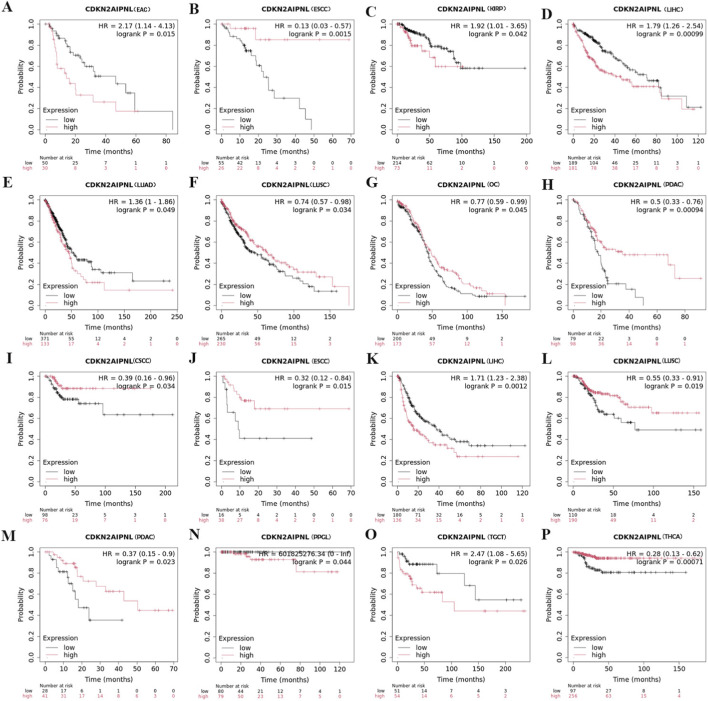
Correlation between the expression of *CDKN2AIPNL* and OS and RFS in patients with different TCGA tumor types in Kaplan-Meier plotter database. **(A–H)** Represents the relationship between *CDKN2AIPNL* expression and OS in different tumors, and **(I–P)** represents the relationship between *CDKN2AIPNL* expression and RFS in different tumors. Survival charts and Kaplan-Meier charts with significant results are displayed. 95% confidence intervals for disease-free survival and overall survival are indicated by red and blue dashed lines for high *CDKN2AIPNL* and low groups, respectively.

In the Kaplan-Meier plotter database, we observed that in LIHC, high *CDKN2AIPNL* expression genes were associated with poorer OS and RFS (n = 370, OS: *HR =* 1.79, *P =* 0.0009; n = 164, DFS: *HR =* 0.39, *P =* 0.034, Figures 3D,K). In Esophageal squamous cell carcinoma (n = 81, OS: *HR =* 0.13, *P =* 0.015; n = 54, RFS: *HR =* 0.32, *P =* 0.015, Figures 3B,J), LUSC (n = 495, OS: *HR =* 0.74, *P =* 0.034; n = 300, RFS: *HR =* 0.55, *P =* 0.019, Figures 3F,L), and PAAD (n = 177, OS: *HR =* 0.5, *P =* 0.000094; n = 69, RFS: *HR =* 0.37, *P =* 0.023, Figures 3H,M), high expression was associated with better OS and RFS. Conversely, in Esophageal adenocarcinoma (n = 80, *HR =* 2.17, *P =* 0.015, Figure 3A), KIRP (n = 287, *HR =* 1.92, *P =* 0.042, Figure 3C), and LUAD (n = 504, *HR =* 1.36, *P =* 0.049, Figure 3E), high *CDKN2AIPNL* expression was associated with poorer OS. In contrast, high expression of *CDKN2AIPNL* was associated with better OS in OV (n = 373, *HR =* 0.77, *P =* 0.045, Figure 3G). Additionally, in PCPG (n = 216, *HR =* 1.71, *P =* 0.0012, Figure 3N) and TGCT (n = 159, *HR =* 0.37, *P =* 0.023, Figure 3O), high *CDKN2AIPNL* expression was associated with poorer OS. Interestingly, in CESC (n = 174, *HR =* 0.39, *P =* 0.034, Figure 3I) and THCA (n = 353, *HR =* 0.28, *P =* 0.00071, Figure 3P), low expression of *CDKN2AIPNL* meant poorer RFS. Collectively, these results indicate that *CDKN2AIPNL* expression is closely associated with the prognosis of various cancer types.

**FIGURE 4:**
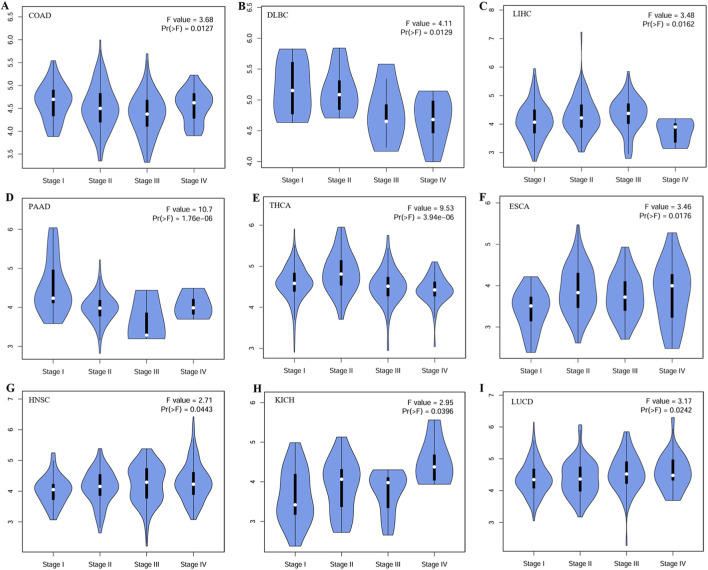
Correlation between *CDKN2AIPNL* expression and tumor pathological staging. The correlation between *CDKN2AIPNL* expression and pathological staging of COAD **(A)**, DLBC **(B)**, LIHC **(C)**, PAAD **(D)**, THCA **(E)**, ESCA **(F)**, HNSC **(G)**, KICH **(H)**, and LUCD **(I)** from the TCGA dataset. Log2 (TPM + 1) was applied on a logarithmic scale.

These results indicate that increased *CDKN2AIPNL* expression is associated with poor prognosis in various tumor types. To further investigate this relationship, we analyzed the correlation between *CDKN2AIPNL* expression and tumor pathological staging using the GEPIA2 database. We observed that low *CDKN2AIPNL* expression was significantly associated with advanced stages of COAD, DLBC, LIHC, PAAD, and THCA, which are cancers with higher metabolism (Figures 4A–E). In contrast, in tumors with lower metabolic rates, *CDKN2AIPNL* expression remained relatively stable across later stages (Figures 4F–I).

### Genetic alterations

**FIGURE 5:**
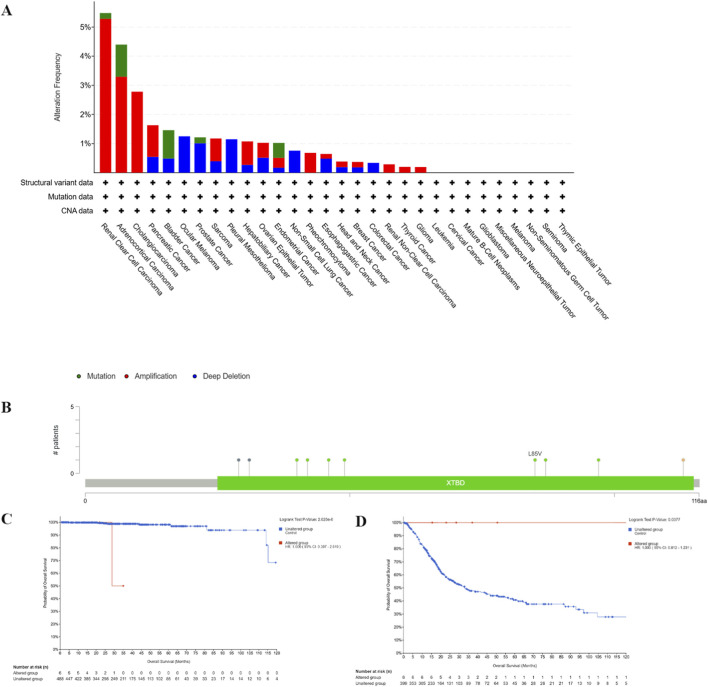
Genetic alterations of *CDKN2AIPNL* in various tumor types of the TCGA. The types of *CDKN2AIPNL* gene alterations **(A)** and the mutation sites of *CDKN2AIPNL* **(B)** were generated by cBioPortal. The correlation between *CDKN2AIPNL* amplification status and PRAD **(C)** OS was analyzed through cBioPortal. The correlation between mutation status and BLCA **(D)** OS was analyzed.

Next, we utilized cBioPortal to examine the genetic alterations of *CDKN2AIPNL* across various tumor types within the TCGA dataset. In KIRC, >5% of tumors harbored *CDKN2AIPNL* amplifications/mutations. (Figure 5A; Supplementary Table S1). In addition, more than 4% of ACC samples showed genetic alterations in *CDKN2AIPNL* (Figure 5A; Supplementary Table S1). As illustrated in Figure 5B, a total of 10 mutations in *CDKN2AIPNL* were detected across TCGA tumor samples, comprising 7 missense mutations, 2 truncating mutations, and 1 splice mutation (Supplementary Table S2). Notably, all mutations occurred within the XTBD region (amino acids 25-115) encoded by *CDKN2AIPNL*, making it the most frequently mutated region of the protein (Figure 5B).

Subsequently, we investigated the association between alterations in the *CDKN2AIPNL* gene and clinical outcomes in cancer patients. Amplifications in PRAD predicted poor OS (*p* = 0.008, Figure 5C; Supplementary Table S3). Conversely, patients with *CDKN2AIPNL* mutated BLCA demonstrated better OS outcomes (Figure 5D; Supplementary Table S4).

### Cancer-associated fibroblast infiltration

**FIGURE 6:**
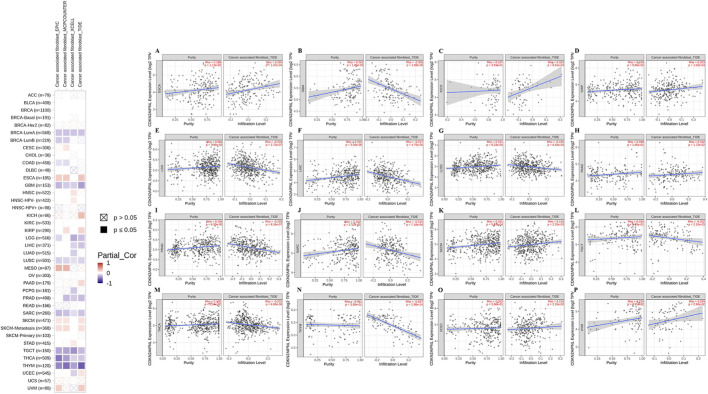
Correlation between *CDKN2AIPNL* expression and cancer-associated fibroblast immune infiltration. **(A–P)** The correlation between *CDKN2AIPNL* expression and cancer-associated fibroblast immune infiltration in all tumor types of the TCGA was calculated using the EPIC and TIDE algorithms.

Previous research has demonstrated that cancer-associated fibroblasts within the tumor stroma play a crucial role in regulating various tumor-infiltrating immune cells (Chen and Song, 2019). Consequently, we employed the EPIC and TIDE algorithms to investigate the correlation between cancer-associated fibroblast infiltration and *CDKN2AIPNL* expression across different malignant tumors. Our analysis revealed a positive correlation between *CDKN2AIPNL* expression and cancer-associated fibroblast infiltration in ESCA (n = 185), KICH (n = 66), KIRP (n = 290), UVM (n = 80), PAAD (n = 179), SKCM-Metastasis (n = 368), SKCM (n = 471), and UCEC (n = 545) (Figure 6).

### *CDKN2AIPNL*-related gene enrichment

**FIGURE 7:**
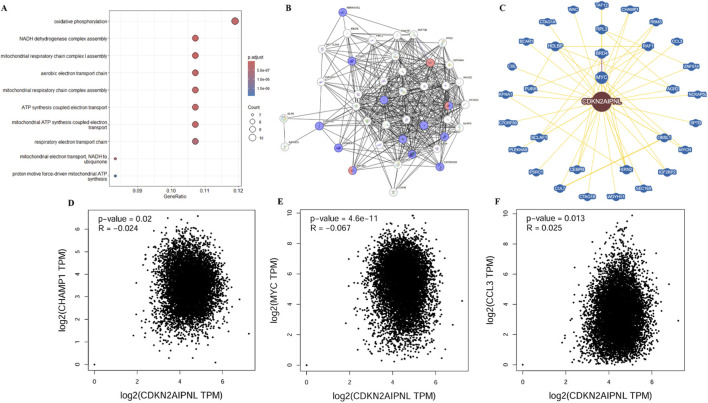
*CDKN2AIPNL*-related gene enrichment analysis. **(A)** GO enrichment analysis of the top 100 genes co-expressed with *CDKN2AIPNL* obtained from GEPIA2. **(B)** Co-expression network of 50 genes obtained with the STRING tool for *CDKN2AIPNL* co-expression. **(C)** *CDKN2AIPNL*-protein interactions obtained through BioGRID. **(D-F)** Correlation analysis between *CDKN2AIPNL* and *CHAMP1*, *XRN2*, and *MYC* from all tumor samples of the TCGA by GEPIA2.

To study the functional mechanism of *CDKN2AIPNL* in oncogenesis, we used GEPIA2 to extract the top 100 genes with similar expression patterns to *CDKN2AIPNL* from all tumor types in the TCGA dataset (Supplementary Table S5). GO enrichment analysis indicated that these genes are related to mitochondrial function and energy metabolism (Figure 7A). Subsequently, 50 genes co-expressed with *CDKN2AIPNL* were identified through the STRING tool to validate the results of the Gene Ontology enrichment analysis. As depicted in Figure 7B, these 50 genes are closely related; moreover, these genes are also enriched in unknown function and Retinal homeobox protein *RAX*/*RAX2* (Supplementary Table S6). These findings led us to investigate the key proteins that *CDKN2AIPNL* may interact with in these biological processes. According to the BioGRID database, *CDKN2AIPNL* physically interacts with *CHAMP1*, *XRN2*, and *MYC* (Figure 7C), all of which are well-characterized genes encoding proteins involved in the regulation of energy metabolism, cell proliferation, differentiation, and tumorigenesis (Supplementary Figure S2). Additionally, we observed that the expression of *CDKN2AIPNL* is closely related to the expression levels of *MYC*, *CHAMP1*, and *XRN2* (Figures 7D–F). Based on these results, we speculate that *CDKN2AIPNL* likely drives tumorigenesis via three axes: (1) *MYC*/*XRN2*-mediated metabolic reprogramming, (2) CAF-dependent immune suppression, and (3) genomic instability via mutation hotspots.

## Discussion

The advancement of gene bioinformatics algorithms, alongside the continuous improvement and updating of databases, has facilitated numerous pan-cancer analyses in recent years aimed at identifying tumor molecular biomarkers and elucidating their functional roles. In this study, we analyzed the prognostic value and oncogenic role of *CDKN2AIPNL* across various tumor types.

*CDKN2AIPNL* is located on chromosome 5q31.1 and encodes two transcripts, both of which produce proteins. Notably, only one transcript, NM_001008278.2, with a length of 1782 nucleotides, generates the 116-amino acid protein NP_001008279.1 (Gerhard et al., 2004). *CDKN2AIPNL* exhibits broad expression in various normal tissues, with the highest expression levels found in the thyroid (*RPKM 3.8*), followed closely by testicular tissue (*RPKM 3.7*) (Fagerberg et al., 2014). According to the Ensembl database, orthologs of *CDKN2AIPNL* have been identified in 489 species. The protein-coding sequence of *CDKN2AIPNL* is relatively conserved among vertebrates. Li-Min Liu et al. found that *CDKN2AIPNL* was significantly elevated in the mechanism of liver cancer occurrence and was associated with poor OS (Liu et al., 2019). Conversely, Visconte et al. identified a tumor suppressor role for *CDKN2AIPNL* in a cohort of 223 myeloid neoplasm cases based on whole-exome sequencing (WES) analyses (Visconte et al., 2017). Additionally, Ali Azimi et al. reported that *CDKN2AIPNL* expression levels were significantly downregulated in melanoma with distant metastasis, suggesting that *CDKN2AIPNL* may function as an inhibitor of distant metastatic processes (Azimi et al., 2023). However, the role of *CDKN2AIPNL* across various tumor types remains inadequately explored. Therefore, we systematically characterized *CDKN2AIPNL* in 33 TCGA tumor types by analyzing gene expression, genetic alterations, and immune infiltration features.

In this study, we observed that *CDKN2AIPNL* is widely expressed in various tissues, and *CDKN2AIPNL* is upregulated in most tumors. We further explored the relationship between *CDKN2AIPNL* overexpression and clinical parameters, including prognosis. Survival analysis shows that the effect of *CDKN2AIPNL* expression level on tumor prognosis is related to tumor type. Currently, research on the prognostic heterogeneity of the *CDKN2AIPNL* gene across different malignancies remains limited, and its underlying molecular mechanisms have not yet been fully elucidated. Future fundamental studies should focus on two key aspects: first, the role of *CDKN2AIPNL* in tumor microenvironment-specific immunoregulatory networks across various cancer types; second, the potential synergistic oncogenic effects between this gene and other somatic mutations. These investigations will provide critical theoretical insights into the tumor biological functions of *CDKN2AIPNL*. Notably, elevated *CDKN2AIPNL* expression correlated with poor prognosis in various tumor types, including Esophageal adenocarcinoma, KIRP, LIHC, LUAD, PCPG, TGCT, THYM, and UVM. Moreover, upregulation of *CDKN2AIPNL* was significantly associated with advanced cancer staging, indicating malignant progression. Increasing evidence suggests that genomic mutations contribute to tumor progression and influence chemotherapy responses (Gu et al., 2023; Chatrath et al., 2021). For instance, Yang et al. reported that mutations in BRCA1 and BRCA2 are significantly associated with patient survival rates, potentially due to unique responses to platinum-based therapy (De Talhouet et al., 2020). In our study, we found that *CDKN2AIPNL* mutations were most prevalent in KIRC (>5%), followed by ACC. In KIRC, *CDKN2AIPNL* has a high frequency of genetic alterations, mainly copy number amplification and missense mutations. These genetic alterations in *CDKN2AIPNL* may potentially influence the response of patients to PD-1/PD-L1 inhibitors, and mutations may lead to the escape of tumor cells from the immune system, which may affect the efficacy of the treatment. In the ACC, mutations in *CDKN2AIPNL* may affect patient response to targeted therapies such as mTOR inhibitors and TKI drugs. Our analysis of the impact of *CDKN2AIPNL* gene alterations on clinical outcomes revealed that *CDKN2AIPNL* amplification may serve as a risk factor for patients with PRAD, while mutations in *CDKN2AIPNL* may confer a protective effect in patients with BLCA. Collectively, these findings suggest that *CDKN2AIPNL* functions as an oncogene in the progression of various cancers and represents a promising predictor for applications in cancer prognosis, despite its previously recognized role as a tumor suppressor gene.

The interaction between immune cells and cancer cells plays a crucial role in shaping the tumor microenvironment and significantly influences the processes of tumor spread and metastasis (Adib et al., 2021; Zhang et al., 2024). Recent studies have reported that the tumor immune microenvironment is associated with the expression levels of various genes (Cai et al., 2024). In our analysis, we found that *CDKN2AIPNL* expression is positively correlated with the infiltration of CAFs in several types. Current evidence indicates that transforming growth factor-beta (TGF-β) and platelet-derived growth factor (PDGF) modulate diverse cellular components within the tumor microenvironment (including T lymphocytes, cancer-associated fibroblasts, and tumor-associated macrophages), thereby fostering an immunosuppressive phenotype and metastatic progression (Manzat Saplacan et al., 2017; Fasano et al., 2024). CDKN2AIPNL may exert either pro-tumorigenic or anti-tumorigenic effects by regulating this microenvironmental remodeling process. Furthermore, another study demonstrated that upregulation of CDKN2A significantly enhances tumor cell proliferation via activation of the JAK2/STAT3 signaling pathway (Yong et al., 2025). CAFs are integral components of the tumor stroma and have been associated with poor prognosis, chemotherapy resistance, and disease recurrence in various cancers (Tajaldini et al., 2022; Chen et al., 2021). *CDKN2AIPNL* interacts with *CDKN2A* to regulate the cell cycle and cell proliferation process (Cao et al., 2022; Shi et al., 2022). *CDKN2A* is an important regulator of the G1 phase of the cell cycle, which inhibits cell proliferation by preventing cells from moving from the G1 phase into the S phase through inhibiting the activity of CDK4/6 (Wang et al., 2024).*CDKN2AIPNL* may indirectly affect immune cells by regulating the function of *CDKN2A* proliferation (Luo et al., 2021). In addition, *CDKN2AIPNL* may also regulate cell proliferation by regulating signaling pathways such as PI3K/Akt and JAK/STAT (Hu et al., 2021). In fact, there are no direct research finding on the effect of *CDKN2AIPNL* on the proliferation of immune cells, but its mechanism of action in cell cycle regulation and signaling pathway modulation suggests that it may affect the proliferation of immune cells through multiple pathways. Further experimental studies are needed to explore the specific function and mechanism of action of *CDKN2AIPNL* in immune cells in order to provide deeper understanding of its role in immune regulation.

In summary, our work elucidates the potential impact of *CDKN2AIPNL* on tumor immunity and its prognostic value across different cancer types.

Using STRING and GEPIA2, we identified numerous genes co-expressed with *CDKN2AIPNL* in various tumors and other tissues. Gene enrichment analysis revealed that these co-expressed genes are closely related to cell cycle regulation, mitochondrial function, and cellular energy metabolism, consistent with findings from previous studies (Cargill et al., 2021; Liang et al., 2024; Li et al., 2022). Furthermore, our results indicate that *CDKN2AIPNL* physically interacts with *MYC*, *CHAMP1*, and *XRN2* (García-Alonso et al., 2022). Importantly, the expression levels of *MYC*, *CHAMP1*, and *XRN2* are closely correlated with *CDKN2AIPNL* expression. These genes are well-characterized and encode proteins that are pivotal in DNA repair, cell cycle regulation, and the regulation of mitochondrial function and cellular energy metabolism (Hino et al., 2021; Chu et al., 2024). Notably, *MYC* is a key transcription factor that plays a significant role in metabolic regulation. Recent studies have shown that *PRMT5* activates lipid metabolic reprogramming via *MYC*, highlighting the importance of *MYC* in metabolic pathways (Liang et al., 2024). This suggests that the co-expression of *CDKN2AIPNL* with *MYC* may contribute to similar metabolic processes. Additionally, *CHAMP1*, which is involved in chromosome segregation and cell cycle regulation, may also influence metabolic regulation through homologous recombination (HR) repair, regulating the expression of the anti-apoptotic protein Mcl-1, and regulating cell division and growth (Li et al., 2022; Hino et al., 2021; Fujita et al., 2022). These findings validate our gene enrichment analysis results and pave the way for further exploration of the molecular functions of *CDKN2AIPNL*.

## Limitations and prospects

This study also has several limitations. First, the sample sizes for some less common tumor types are relatively small, potentially leading to batch effects or inaccuracies in the results. Second, while this study provides preliminary insights linking *CDKN2AIPNL* to the progression of various tumors. Nevertheless, systematic functional experiments (Such as CRISPR-Cas9-mediated knockout, Western blot analysis, and cell cycle profiling) are still required to delineate the precise molecular mechanisms of CDKN2AIPNL in tumorigenesis and progression. Finally, Liu’s laboratory was one of the first laboratories involved in TCGA biomarker research and has developed various strategies in this field (Liu et al., 2024a; Li and Liu, 2022; Liu et al., 2024b). The broad and diverse nature of TCGA data makes it an important resource for cancer research, but there are limitations such as sample selection bias and tissue heterogeneity, which may affect the generalizability of the results (Liu et al., 2024c). Therefore, further basic studies need to be designed for the Therefore, further basic studies need to be designed to validate the relevant findings.

In conclusion, *CDKN2AIPNL* is widely overexpressed in various cancers, and both its expression and genetic alterations are statistically correlated with the clinical outcomes of affected patients. Moreover, immune infiltration analysis and *CDKN2AIPNL*-related gene enrichment analyses offer potential mechanisms through which *CDKN2AIPNL* may regulate tumor immunity, cell cycle dynamics, mitochondrial function, and cellular energy metabolism in cancer. Therefore, additional experimental and clinical studies are warranted to explore the practical applications of *CDKN2AIPNL* in cancer treatment and prognosis prediction.

## Data Availability

The datasets generated and/or analysed during the current study are at the following website: HPA: https://www.proteinatlas.org/; TIMER2: http://timer.cistrome.org/; GEPIA2: http://gepia2.cancer-pku.cn/; the UCSC Xena browser: https://xenabrowser.net/; cBioPortal: https://www.cbioportal.org/; The STRING tool: https://string-db.org/; BioGRID: https://thebiogrid.org/.
