# Supplementary figures and images for: CDKN2AIPNL: a potential pan-cancer biomarker
Source: Front Genet. 2026 Jan 21;16:1588292. doi: 10.3389/fgene.2025.1588292 (PMC12867337; doi:10.3389/fgene.2025.1588292)

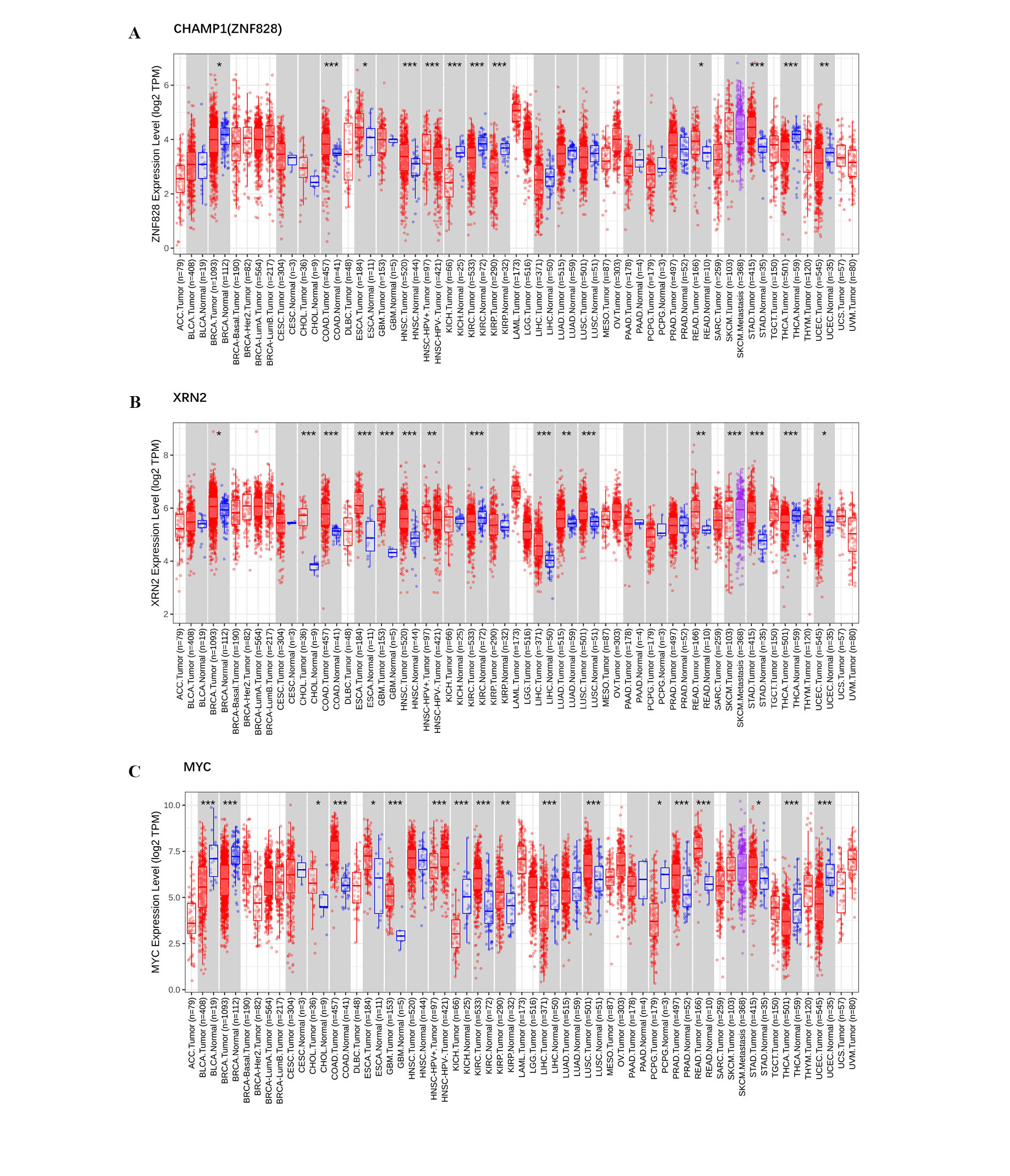

Supplement: Supplementary file 1 [file Image2.png]

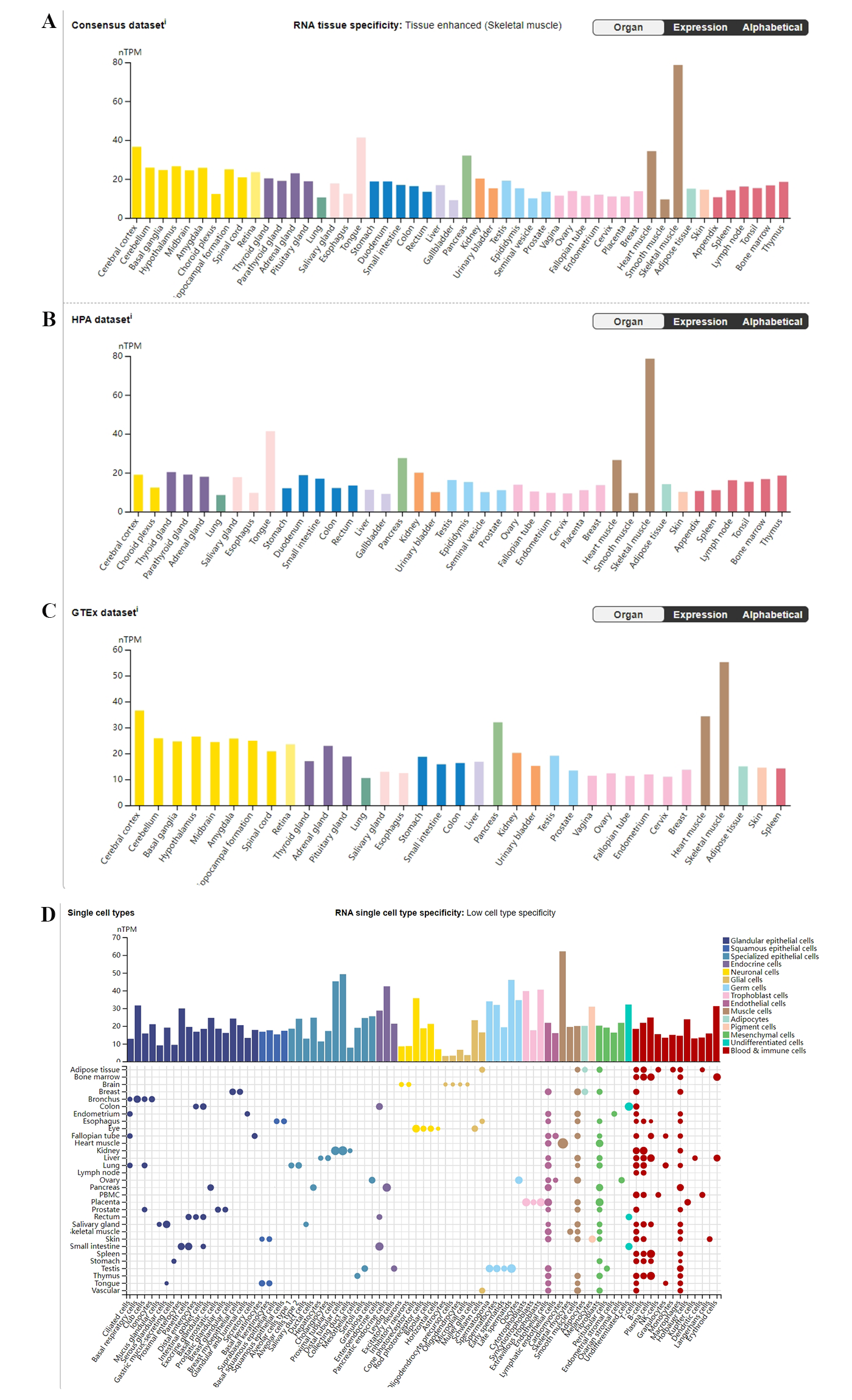

Supplement: Supplementary file 3 [file Image1.png]
